# The Influence of Disease upon Population

**Published:** 1887-08

**Authors:** 


### The Influence of Disease upon Population.

In a very interisting paper by Walter A. Dun, M.D., published in the *Cincinnati Lancet and Clinic*, the author says:

The United States has doubled its population every twenty-five years for over two hundred and twenty-five years. If this rate continues for another century, the population will exceed in density that of the Chinese Empire. From 7,860,000 in 1820 it has risen to about 60,000,000 in 1886. It requires only a little application of these figures to show that all known checks to the natural increase of population must be actively at work in a few years in order to keep down the results of procreation. The advance in pathology warrants the belief that disease as a factor in the reduction of population will be less potent in the future than heretofore, and other well-recognized checks are likely to be more potent.

*Want* is a check which constantly appears in our path, from improvidence, intemperance, misfortune, or other reason, and as constantly lifts its head in appeal to our kinder instincts. No one can deny its marked increase in densely crowded cities. Our country produces food sufficient for at least 20,000,000 people more than its present population, and yet *want* is a check to her population, a check which is constantly growing, and which must be of more consequence as our food supply nears the point of just supplying the necessities of our own people.

The procreative instinct is one of the most powerful which exists in all forms of life, both animal and vegetable. This strong law of nature is one from which man vainly tries to escape. By moral restraint, prevention of conception and abortion, the reproduction of the species is limited by man, and he seeks by these ways to escape the strife of over-population. The struggle to exist must make them prominent in that dense population to which we are rapidly drifting. There are strong reasons for believing that long before we reach a population as dense as that predicted for this country fifty years hence, an armed struggle, within or without, will offer to surgeons unusual opportunities. Intemperance as a check to population can hardly be estimated now. The trial of prohibition is spreading among the people of the United States. Viewed in the light of the past, all must see that the advance made in pathology during the last few years is not an unmixed good, but that it throws its savings into other channels, and is compensated for in other ways.

